# Functional skeletal muscle constructs from transdifferentiated human fibroblasts

**DOI:** 10.1038/s41598-020-78987-8

**Published:** 2020-12-16

**Authors:** Bin Xu, Allison Siehr, Wei Shen

**Affiliations:** 1 Department of Biomedical Engineering, University of Minnesota, Minneapolis, MN 55455 USA; 2 Stem Cell Institute, University of Minnesota, Minneapolis, MN 55455 USA; 3 Institute for Engineering in Medicine, University of Minnesota, Minneapolis, MN 55455 USA

**Keywords:** Biomedical engineering, Tissue engineering

## Abstract

Transdifferentiation of human non-muscle cells directly into myogenic cells by forced expression of MyoD represents one route to obtain highly desirable human myogenic cells. However, functional properties of the tissue constructs derived from these transdifferentiated cells have been rarely studied. Here, we report that three-dimensional (3D) tissue constructs engineered with iMyoD-hTERT-NHDFs, normal human dermal fibroblasts transduced with genes encoding human telomerase reverse transcriptase and doxycycline-inducible MyoD, generate detectable contractile forces in response to electrical stimuli upon MyoD expression. Withdrawal of doxycycline in the middle of 3D culture results in 3.05 and 2.28 times increases in twitch and tetanic forces, respectively, suggesting that temporally-controlled MyoD expression benefits functional myogenic differentiation of transdifferentiated myoblast-like cells. Treatment with CHIR99021, a Wnt activator, and DAPT, a Notch inhibitor, leads to further enhanced contractile forces. The ability of these abundant and potentially patient-specific and disease-specific cells to develop into functional skeletal muscle constructs makes them highly valuable for many applications, such as disease modeling.

## Introduction

Even though normal skeletal muscle has self-regenerative ability, it has remained a major challenge to treat genetic muscle diseases and volumetric muscle loss^1^. Using human myogenic cells to develop cell-based therapies to repair or restore diseased or lost muscle tissue represents an important strategy to address this challenge. Human myogenic cells are also highly desirable for creating muscle tissue models or disease models, which can be used for fundamental studies of muscle physiology and pathology and for drug screening and validation. Many studies have used animal myogenic cells, such as the C2C12 cell line and myoblasts isolated from animal models of muscular dystrophy, to create muscle tissue models or disease models. These studies have made significant contributions to the research area, but striking interspecies differences make animal cells unable to provide reliable models for many human genetic diseases^2–5^.

Despite their importance, studies using human myogenic cells have been hampered by insufficient cell quantity. Traditionally, human myogenic cells have been obtained from muscle biopsies, which have limited availability, and the cells have poor expansion capacity^6^. Generation of immortalized human myoblasts is technically challenging. Transduction of human myoblasts with human telomerase reverse transcriptase (hTERT) is not sufficient to immortalize these cells^7^. Even though overexpression of cyclin-dependent kinase 4 (CDK4) or cyclin D1 with hTERT could result in myoblasts capable of extensive proliferation, these cells have compromised myogenic differentiation potential^7,8^. Recent advances in deriving myogenic cells from induced human pluripotent stem cells (hiPSCs) may provide opportunities to address the limited availability of human myogenic cells^9–12^. Alternatively, transdifferentiation, which is direct conversion of one somatic cell type to another without going through the pluripotent state^13^, may offer another approach to obtaining human myogenic cells. In both methods, somatic cells are used as the starting cells, making it possible to obtain abundant, patient-specific, and disease-specific human myogenic cells for therapeutic or disease modeling purposes.

Direct conversion of fibroblasts into myogenic cells by forced expression of the transcription factor MyoD was first demonstrated about three decades ago^14^, representing the earliest example of transdifferentiation. Since then, it has been shown that MyoD-induced transdifferentiation can convert many other cell types into myogenic cells^15^, though fibroblasts remain the most commonly used starting cell source. Human myogenic cells obtained through transdifferentiation have several advantages over those derived from hiPSCs: direct conversion of one somatic cell type to the target lineage is substantially quicker than reprograming the starting cell type to hiPSCs and then further guiding differentiation toward the target lineage; transdifferentiated cells avoid the risk of teratoma formation associated with pluripotent stem cells^16^. However, the efficiency of myogenic transdifferentiation needs to be improved, and literature information on functional evaluation, such as contractile force generation, of these cells is scarce.

In this study, we established a cell line through viral transduction of primary normal human dermal fibroblasts (NHDFs) with genes encoding hTERT and doxycycline (DOX) inducible MyoD (iMyoD). The hTERT gene was introduced into the cells to enhance their expansion capacity. NHDFs were chosen because they can be obtained through a minimally invasive procedure. We examined the abilities of these cells to proliferate and to transdifferentiate into myogenic cells upon DOX-induced MyoD expression. We investigated the roles of two small molecules CHIR99021 (a Wnt signaling activator) and DAPT (a Notch signaling inhibitor) in enhancing functional myogenic differentiation of these iMyoD-converted fibroblasts. We further engineered three-dimensional (3D) tissue constructs with these cells and examined their ability to generate contractile forces in response to electrical stimuli. The effects of temporal modulation of MyoD expression and treatment with CHIR99021 and DAPT on contractile force generation of these 3D constructs were also investigated.

## Results and discussion

### Generate iMyoD-hTERT-NHDFs and primary iMyoD-NHDFs

**Figure 1:**
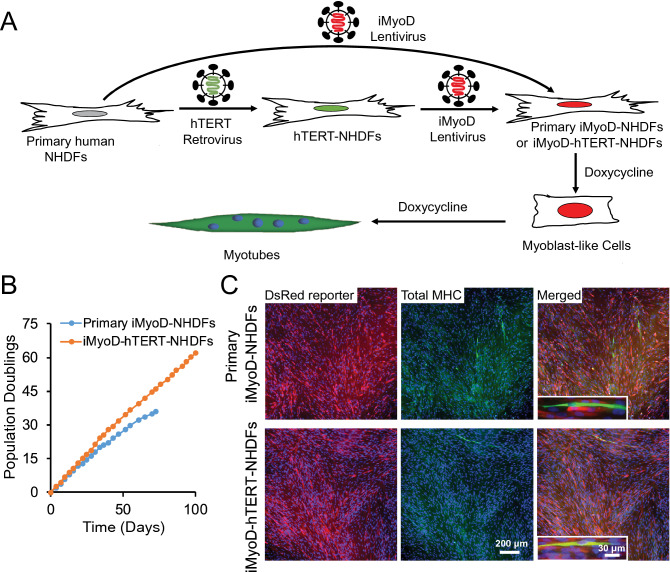
Generation of primary iMyoD-NHDFs and iMyoD-hTERT-NHDFs and their myogenic transdifferentiation via induced MyoD expression. (**A**) Illustration of the procedures to generate these cells through viral transductions and to induce MyoD expression with DOX. (**B**) The extensive expansion capacity of iMyoD-hTERT-NHDFs endowed by hTERT as indicated by an almost unchanged growth rate at high population doubling values. (**C**) Representative images showing that both cell types expressed the DsRed reporter for iMyoD (red) and cells positive for total MHC (green) were present after 3-day DOX induction. The total MHC was stained with the MF20 antibody, and nuclei were stained with Hoechst 33342 (blue).

We set out to generate iMyoD-NHDFs by lentivirally transducing primary NHDFs with a Tet-On VP64-MyoD (iMyoD) vector as previously reported (Fig. 1A)^17^. This vector allows constitutive co-expression of a puromycin resistance gene for selection of transduced cells and a reverse tetracycline transactivator for doxycycline (DOX) inducible expression of MyoD and a red fluorescent reporter DsRed; the transcriptional activator VP64 fused to MyoD enhances chromatin remodeling and benefits transdifferentiation^17^. To address the limited proliferative capacity of primary iMyoD-NHDFs, we further generated an immortalized iMyoD-hTERT-NHDF cell line (Fig. 1A). An immortalized NHDF cell line (hTERT-NHDFs) was generated by transducing primary NHDFs with a vector encoding hTERT to overcome the Hayflick limit in primary cell expansion^18^, then the hTERT-NHDFs were transduced with the iMyoD vector to obtain iMyoD-hTERT-NHDFs. The presence of the transduced genes in the genomic DNA of these cells was confirmed by PCR analysis (Figure S1). To confirm the robustness of the transdifferentiation method, primary NHDFs from two donors were used, and the conclusions in this paper are supported by the cell lines derived from both primary NHDFs.

Before DOX induction, both iMyoD-hTERT-NHDFs and primary iMyoD-NHDFs displayed normal fibroblast morphology. To test their proliferation capacity, the cells were seeded in 48-well plates (2 × 10^4^ cells in each well) and cultured in an NHDF culture medium, and the population doubling as a function of time was measured. The iMyoD-hTERT-NHDFs exhibited prolonged and stable proliferation capacity, with an almost unchanged growth rate even when the population doubling value was greater than 60 (Fig. 1B). In contrast, primary iMyoD-NHDFs showed gradually reduced growth rates, and the growth became very slow when the population doubling value reached approximately 30 (Fig. 1B). These results confirm that hTERT transduction allows the cells to overcome their expansion limit, providing an abundant source of human myogenic cells.

### The myogenic differentiation efficiency upon DOX induction is low

When iMyoD-hTERT-NHDFs and primary iMyoD-NHDFs were seeded at approximately 20% confluency and cultured in the NHDF culture medium supplemented with DOX for 3 days, both cell types exhibited similar myogenic differentiation tendency, as indicated by elongated cell morphology and positive immunofluorescence staining with the MF20 antibody for total myosin heavy chains (total MHC), a characteristic marker for skeletal muscle (Fig. 1C). However, even after 7-day DOX induction, the myotubes were still short and the myogenic index (defined as the ratio of the nuclei number in the MF20^+^ cells containing three or more nuclei to the total number of nuclei)^19^ was low (Figure S2A), indicative of retarded myogenic differentiation. Cells at this stage were proliferative and could be passaged or cryopreserved with their proliferative and myogenic capacities retained. These results suggest that DOX-induced MyoD expression can guide iMyoD-hTERT-NHDFs and primary iMyoD-NHDFs to become myoblast-like cells, but it is insufficient to efficiently drive terminal myogenic differentiation of these myoblast-like cells.

**Figure 2:**
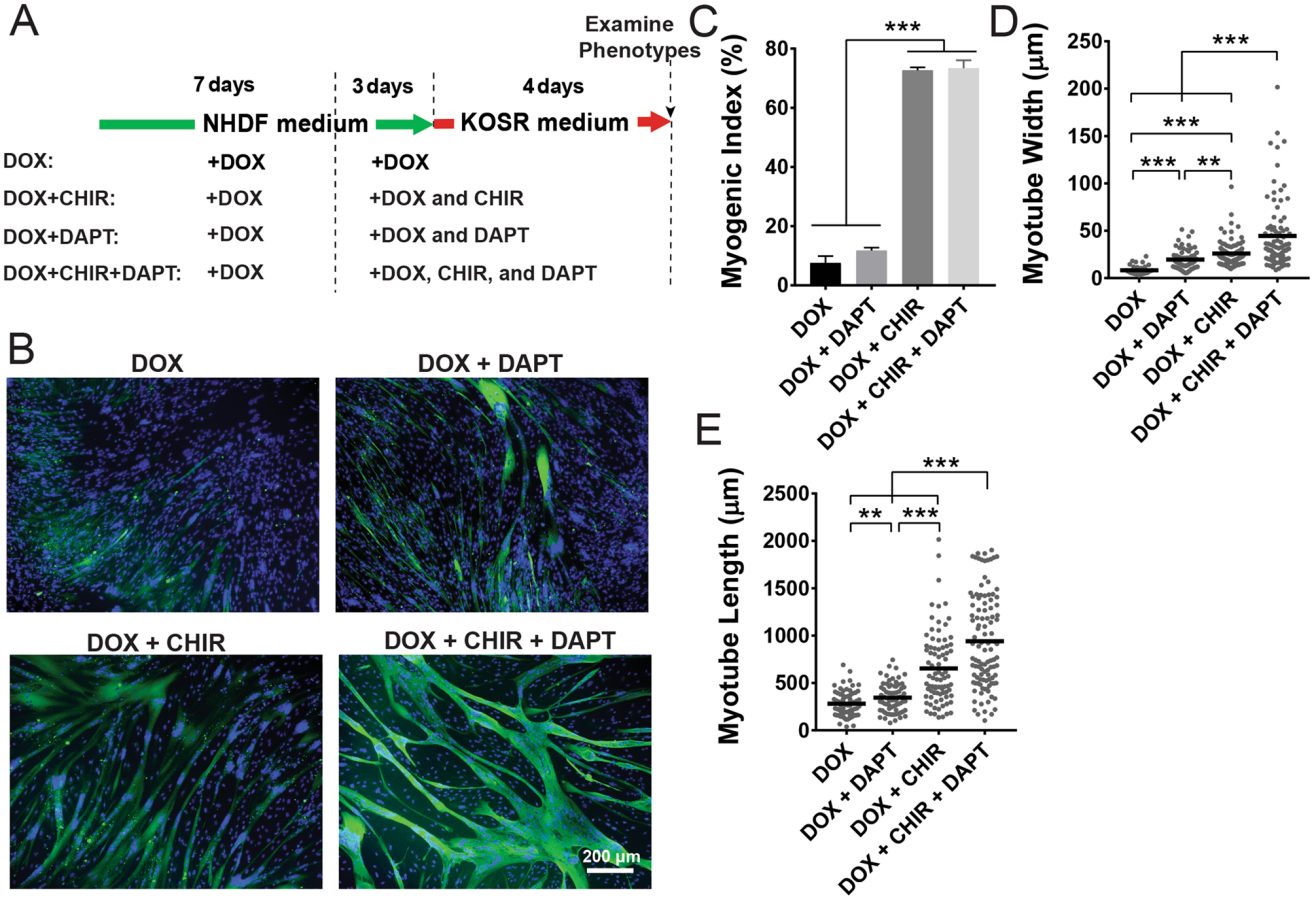
Effects of CHIR99021 and DAPT on myogenic differentiation of iMyoD-hTERT-NHDFs in 2D culture. (**A**) Illustration of cell culture regimens. (**B**) Cell morphology after 2 weeks of culture as revealed by immunofluorescent staining for total MHC (green) and counterstaining for nuclei (blue). (**C**–**E**) Quantification of myogenic index, myotube width, and myotube length. The experiments were conducted in triplicate with 3 independent samples. Data are presented as mean ± SEM. Statistical analysis was performed by one-way ANOVA with Tukey’s test. **p < 0.01, ***p < 0.001.

It is known that normal myogenesis involves myogenic lineage commitment and terminal differentiation^20^, so we reasoned that conversion of iMyoD-hTERT-NHDFs or primary iMyoD-NHDFs to muscle cells could also be divided into these two stages. We expected that a two-stage cell culture regimen, in which the first stage allows myogenic lineage commitment driven by MyoD expression and the second stage promotes terminal myogenic differentiation of the transdifferentiated MyoD^+^ myoblast-like cells, would benefit the overall efficiency of myogenic differentiation. It had been reported that terminal differentiation of myogenic cells requires a low serum condition and high cell density^21^. Therefore, we expected that the efficiency of terminal myogenic differentiation would be improved in the second stage by replacing the serum-rich NHDF medium (containing 20% FBS) with the low-serum KOSR medium, which had been used in myogenic differentiation of hPSC-derived progenitor cells^9,12^. The iMyoD-hTERT-NHDFs were cultured using a 2-week culture regimen (the DOX condition in Fig. 2A) in which the cells were cultured in DOX-containing NHDF medium during the first 10 days to allow lineage commitment and cell expansion and then cultured in DOX-containing KOSR medium in the last 4 days to promote terminal differentiation. However, not much improvement in myogenic differentiation was observed (Fig. 2B) as compared with the cells cultured in DOX-containing NHDF medium for 7 days (Figure S2A).

### CHIR99021 treatment promotes myogenic differentiation of transdifferentiated myoblast-like cells

It has been reported that the small molecule CHIR99021 (abbreviated as CHIR), a GSK-3β inhibitor that activates the canonical Wnt signaling pathway, enhances myogenic differentiation of iMyoD-converted primary human and mouse fibroblasts^22,23^. To examine whether CHIR could promote myogenic differentiation of iMyoD-converted immortalized human fibroblasts, iMyoD-hTERT-NHDFs were expanded for 7 days in DOX-containing NHDF medium, re-plated at a density of 6 × 10^4^ cells/cm^2^, cultured in DOX-containing NHDF medium supplemented with 5 µM CHIR for 3 days, and then cultured in DOX-containing KOSR medium supplemented with CHIR for 4 days (the DOX + CHIR condition in Fig. 2A). These cells showed substantially enhanced myogenic differentiation as compared with those cultured in the DOX condition: the myogenic index increased over ninefold from 7.8 ± 2.3 to 72.7 ± 1.0%; the myotube width and length increased from 8.3 ± 4.5 to 25.9 ± 13.0 µm and from 280.5 ± 134.0 to 653.5 ± 383.0 µm, respectively (Fig. 2B–E). Even though the measurement of myotube length involves manually drawing a line along a myotube in ImageJ and therefore human bias cannot be completely eliminated, the increase in myotube length upon addition of CHIR was significant as shown in Fig. 2B. In the CHIR-containing conditions, many myotubes extended beyond the imaging frame, and the myotube lengths for these conditions are actually underestimated here. In the absence of MyoD expression, CHIR alone could not drive myogenic differentiation, as a control cultured for 7 days in DOX-free NHDF medium supplemented with CHIR did not form any MF20^+^ myotubes (Figure S2B). These results suggest that CHIR alone could not myogenically convert iMyoD-hTERT-NHDFs, but it could enhance myogenic differentiation of MyoD-converted, myoblast-like cells to form multinucleated myotubes.

### Myogenic differentiation of transdifferentiated myoblast-like cells is substantially enhanced by CHIR99021 and DAPT co-treatment

We hypothesized that another small molecule DAPT, a γ-secretase inhibitor that reduces Notch activity, could contribute to myogenic differentiation of transdifferentiated myoblast-like cells and co-treatment with CHIR and DAPT, in conjunction with MyoD expression, would further enhance myogenic differentiation. This hypothesis was formulated based on the literature showing that DAPT can effectively enhance the terminal differentiation of myoblasts in vivo and in vitro^24,25^ and that CHIR and DAPT together can enhance the efficiency of deriving myogenic progenitors from hiPSCs^26^. To the best of our knowledge, the use of DAPT to support myogenic differentiation of transdifferentiated, myoblast-like cells has never been reported.

To test this hypothesis, iMyoD-hTERT-NHDFs were expanded for 7 days in DOX-containing NHDF medium and re-plated at a density of 6 × 10^4^ cells/cm^2^. The cells were then cultured in DOX-containing NHDF medium for 3 days and in DOX-containing KOSR medium for 4 days sequentially, with both media supplemented with DAPT (the DOX + DAPT condition in Fig. 2A) or with CHIR and DAPT (the DOX + CHIR + DAPT condition in Fig. 2A). The myogenic index in the DOX + DAPT condition was 11.75 ± 1.0%, slightly greater than, but not statistically different from, that in the DOX condition (p = 0.1735) (Fig. 2B,C). The myogenic index in the DOX + CHIR + DAPT condition was 73.4 ± 2.7%, substantially greater than that in the DOX + DAPT condition (increased over sixfold), but similar to that in the DOX + CHIR condition (Fig. 2B,C). These results revealed that addition of DAPT in culture media did not enhance the myogenic index; in contrast, the presence of CHIR in the DOX + CHIR and DOX + CHIR + DAPT conditions led to approximately ninefold and sixfold increases in the myogenic index when compared with the DOX and DOX + DAPT conditions, respectively. Since the myogenic index is a morphological marker for the commitment for terminal myogenic differentiation^27^, these results suggest that CHIR, but not DAPT, is a potent promoter for the commitment for terminal myogenic differentiation of these transdifferentiated myoblast-like cells.

The myotube width and length in the DOX + DAPT condition were 19.6 ± 1.3 µm and 345.7 ± 17.5 µm, respectively, significantly greater than those in the DOX condition (width 8.3 ± 4.5 µm; length 280.5 ± 134.0 µm) (Fig. 2B,D,E). The myotube width and length in the DOX + CHIR + DAPT condition were 44.5 ± 3.9 µm and 940.5 ± 47.41 µm, respectively, substantially greater than those in the DOX + CHIR condition (width 25.9 ± 13.0 µm; length 653.5 ± 383.0 µm) (Fig. 2B,D,E). The increases in myotube width and length in the presence of DAPT suggest that it promotes terminal myogenic differentiation by driving committed cells to enter a more advanced differentiation stage as it does in normal myogenesis. Notably, the morphology of the wide and long myotubes in the DOX + CHIR + DAPT condition resembles that of primary human skeletal myotubes^28^.

The DOX + CHIR + DAPT condition illustrated in Fig. 2A was used to verify myogenic transdifferentiation capacity of iMyoD-hTERT-NHDFs after extensive expansion. The iMyoD-hTERT-NHDFs expanded by 15 successive passages after establishment of the cell line were used in the experiment. Multinucleated, MF20^+^ myotubes with wide, long, and fused morphology were observed (Figure S3), similar to those transdifferentiated from iMyoD-hTERT-NHDFs at low passage numbers (Fig. 2B). These results suggest that the extensive expansion capacity of iMyoD-hTERT-NHDFs endowed by hTERT does not sacrifice their myogenic transdifferentiation potential. Preservation of the myogenic transdifferentiation potential at high passage numbers makes iMyoD-hTERT-NHDFs an unlimited source of human myogenic cells for a variety of applications, such as disease modeling^29^.

### CHIR99021 and DAPT have different roles in enhancing myogenic differentiation of transdifferentiated myoblast-like cells

**Figure 3:**
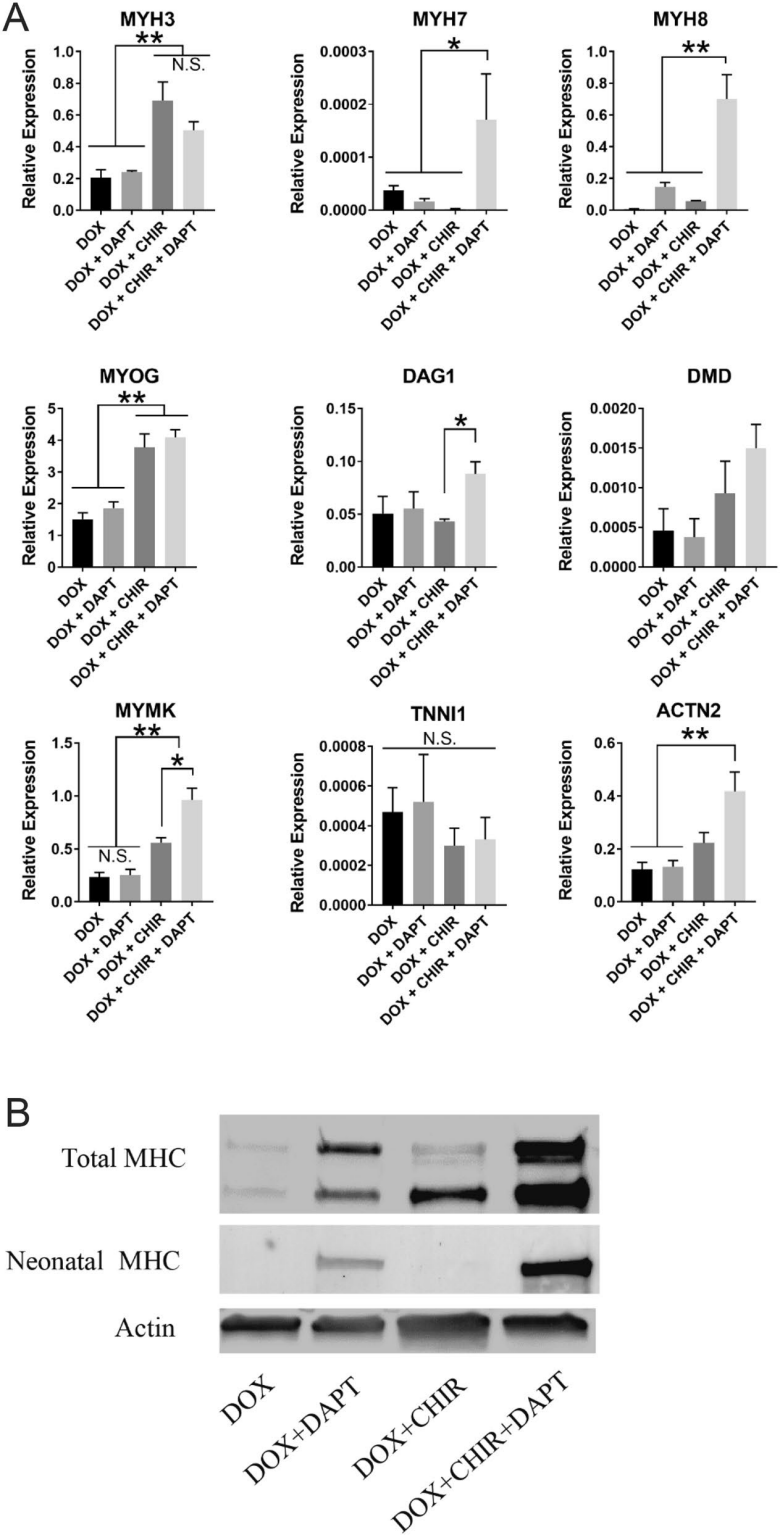
Gene expression and western blot analyses for iMyoD-hTERT-NHDFs cultured using the culture regimens illustrated in Fig. 2A. (**A**) Relative gene expression analysis for myogenic markers. The expression levels were normalized to a housekeeping gene β-actin. The experiments were conducted in triplicate with 3 independent samples. Data are presented as mean ± SEM. Statistical analysis was performed by one-way ANOVA with Tukey’s test. *p < 0.05, **p < 0.01. (**B**) Western blot analysis of total MHC and neonatal MHC. Actin was used as a housekeeping protein. Full-length blots are presented in Figure S5.

To further examine the roles of CHIR and DAPT during myogenic differentiation of transdifferentiated myoblast-like cells, iMyoD-hTERT-NHDFs were cultured using the 4 culture regimens illustrated in Fig. 2A and expressions of characteristic genes for skeletal muscle were characterized using quantitative RT-PCR (Fig. 3A). These genes included myogenic regulatory factor myogenin (MYOG), muscle fusion regulator myomaker (MYMK), muscle structural proteins (including α-actinin (ACTN2), dystrophin (DMD), and laminin-binding protein dystroglycan (DAG1)), as well as contractile and regulatory proteins (including myosin heavy chain (MHC) isoforms (MYH3, 7, 8) and troponin I (TNNI1)).

Expression of MYOG was tremendously upregulated in the CHIR-containing conditions (DOX + CHIR and DOX + CHIR + DAPT) as compared with that in the CHIR-free conditions (DOX and DOX + DAPT). The presence of CHIR also upregulated MYH3 (embryonic myosin heavy chain) and MYMK, two downstream targets of MYOG^30,31^. The upregulation of the fusion regulator MYMK in the CHIR-containing conditions was consistent with the better fused cell morphology as shown in Fig. 2B and with the increased myotube width and length as shown in Fig. 2D,E, as it is known that MYMK upregulation contributes to the formation of long and wide myotubes^32^. Since MYOG expression is a marker for the commitment for terminal myogenic differentiation of myoblasts^33,34^, these results suggest that CHIR promotes the commitment of transdifferentiated myoblast-like cells for terminal myogenic differentiation, consistent with the results of the myogenic index characterization as shown in Fig. 2C.

Addition of DAPT in the culture regimens did not increase the gene expression of MYOG or MYH3, suggesting that DAPT does not promote transdifferentiated myoblast-like cells to commit to terminal myogenic differentiation, consistent with the results of the myogenic index characterization as shown in Fig. 2C. However, DAPT upregulated many muscle-specific markers expressed later in terminal myogenic differentiation. Cells cultured in the DOX + CHIR + DAPT condition exhibited substantially elevated gene expressions of MYH7 (adult slow myosin heavy chain) and MYH8 (neonatal myosin heavy chain), two contractile proteins indicating more advanced differentiation statuses than embryonic MYH3. These cells also showed significantly upregulated gene expression of DAG1 and slightly increased gene expressions of DMD and ACTN2 as compared with those cultured in the DOX + CHIR condition (DAG1, DMD, and ACTN2 encode proteins associated with the contractile machinery in muscle). These results suggest that DAPT upregulates the proteins forming or associating with the contractile machinery, a sign of the cells entering a more advanced stage of terminal myogenic differentiation. Therefore, the gene expression analysis also suggests that DAPT contributes to myogenic differentiation of transdifferentiated myoblast-like cells by driving the cells already committed to terminal differentiation to further differentiate and mature, consistent with the results of the myotube morphology characterization as shown in Fig. 2D,E. The detectable expression of TNNI1 further confirmed the conversion of the fibroblasts to muscle cells.

To further confirm the roles of DAPT and CHIR in modulating myogenic differentiation of transdifferentiated myoblast-like cells, we assessed their effects on primary iMyoD-NHDFs cultured using the culture regimens illustrated in Fig. 2A. Quantitative PCR analysis of MYH3, MYH7, MYH8, and MYMK gene expressions for the cells cultured in the DOX, DOX + DAPT, DOX + CHIR, and DOX + CHIR + DAPT conditions revealed similar trends to those for iMyoD-hTERT-NHDFs (Figure S4).

The roles of CHIR and DAPT in myogenic differentiation of transdifferentiated myoblast-like cells were further analyzed by western blot. Both CHIR and DAPT significantly elevated protein expression of total MYH, as shown by the much higher expression levels in the DOX + DAPT, DOX + CHIR, and DOX + CHIR + DAPT conditions than that in the DOX condition (Fig. 3B, the multiple bands were attributed to MYH isoforms simultaneously blotted by the antibody MF20^35^). The substantially higher expression of total MYH in the DOX + CHIR + DAPT condition as compared with those in the DOX + DAPT and DOX + CHIR conditions suggests that CHIR and DAPT enhance myogenic differentiation of transdifferentiated myoblast-like cells synergistically. Blotting for neonatal MYH revealed negligible protein expression in the DAPT-free conditions and substantially enhanced protein expression when DAPT was added (Fig. 3B), suggesting that DAPT, but not CHIR, promotes muscle maturation of these cells. The western blot results are consistent with the gene expression data shown in Fig. 3A. Notably, the cells cultured in the DOX + CHIR + DAPT condition displayed the most intense bands for both total MYH and neonatal MYH, further confirming that co-treatment with CHIR and DAPT can significantly enhance the efficiency of myogenic differentiation of transdifferentiated myoblast-like cells.

It is known that CHIR activates the canonical Wnt signaling and DAPT inhibits Notch 1 signaling, so the modulations of myogenic differentiation of transdifferentiated myoblast-like cells by these two small molecules most likely relates to Wnt and Notch signaling pathways. It has been reported that orchestration of Notch and Wnt signaling is required for normal myogenesis^36,37^. Activation of the canonical Wnt signaling upregulates myogenic regulatory factors, including MYOG, to promote the commitment for terminal myogenic differentiation^38^, and inhibition of Notch signaling further drives the differentiation and maturation of the committed cells^24,25,39^. Our results show that co-treatment with the Wnt activator CHIR and the Notch inhibitor DAPT significantly enhanced myogenic differentiation of these cells. Furthermore, CHIR primarily enhanced the commitment for terminal myogenic differentiation, while DAPT mainly contributed to promoting further differentiation and maturation of the committed cells. Therefore, the roles of Wnt and Notch modulations in myogenic differentiation of transdifferentiated myoblast-like cells are similar to those in normal myogenesis.

### Temporally controlled MyoD expression benefits force generation of tissue constructs derived from transdifferentiated myoblast-like cells

**Figure 4:**
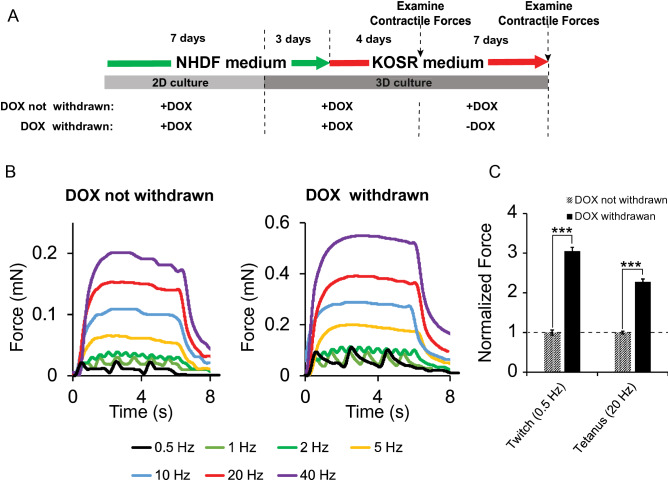
Contractile force generation by 3D tissue constructs engineered with iMyoD-hTERT-NHDFs. (**A**) Illustration of cell culture regimens. (**B**) Representative contractile force patterns generated by the constructs cultured using the two regimens illustrated in (**A**). (**C**) Temporally controlled, rather than prolonged, MyoD expression benefited as revealed from the comparison of the contractile forces. Forces were normalized to the condition in which DOX was withdrawn in the second week of 3D culture. The experiments were conducted in triplicate with 3 independent samples. Data are presented as mean ± SEM. Statistical analysis was performed by t-test. ***p < 0.001.

Contractile force generation in response to electrical stimulation is an essential function of skeletal muscle. We used a tissue engineering approach to create 3D constructs with iMyoD-hTERT-NHDFs and assessed their contractile force generation in response to external electrical stimulation. The iMyoD-hTERT-NHDFs were first cultured in DOX-containing NHDF medium two-dimensionally (2D) for 7 days, trypsinized, encapsulated in a hydrogel composed of fibrin and Matrigel, and cast in a homemade culture device as we reported previously^40^. The 3D constructs were further cultured in DOX-containing NHDF medium for 3 days, followed by culture in DOX-containing KOSR medium for 4 days (Fig. 4A). Functional examination at this time point showed no detectable contractile forces (7 days of 3D culture and the first time point to evaluate force generation as illustrated in Fig. 4A, corresponding to the time point at which phenotypes were examined for 2D culture as illustrated in Fig. 2A). When we extended the culture in DOX-containing KOSR medium for an additional 7 days, contractile forces were detected, indicating that the myogenic cells transdifferentiated from human fibroblasts via forced MyoD expression can develop into functional skeletal muscle constructs. The force patterns generated by these constructs were similar to those generated by the 3D constructs engineered with hPSC-derived myogenic progenitors (Fig. 4B)^40^.

We reasoned that termination of MyoD expression in the middle of myogenic differentiation would promote these 3D constructs to enter a functionally more advanced stage and generate greater contractile forces. This hypothesis was based on the fact that MyoD, an early regulatory factor for myogenic lineage commitment and for the onset of terminal myogenic differentiation, diminishes with myotube maturation during muscle development^20^. To test this hypothesis, we withdrew DOX from the KOSR medium during the second week of 3D culture (Fig. 4A). This change resulted in increases of 3.05 and 2.28 times in the twitch (at 0.5 Hz) and tetanic (at 20 Hz) forces, respectively (Fig. 4B,C). These results suggest that the role of MyoD in myogenic differentiation of transdifferentiated myoblast-like cells is similar to that in normal myogenesis: its expression is essential for myogenic lineage commitment of iMyoD-hTERT-NHDFs and the onset of terminal myogenic differentiation of the committed myoblast-like cells, but prolonged MyoD expression hampers the progression of the terminal differentiation toward cells having contractile properties.

### CHIR99021 and DAPT co-treatment enhances force generation of tissue constructs derived from transdifferentiated myoblast-like cells

**Figure 5:**
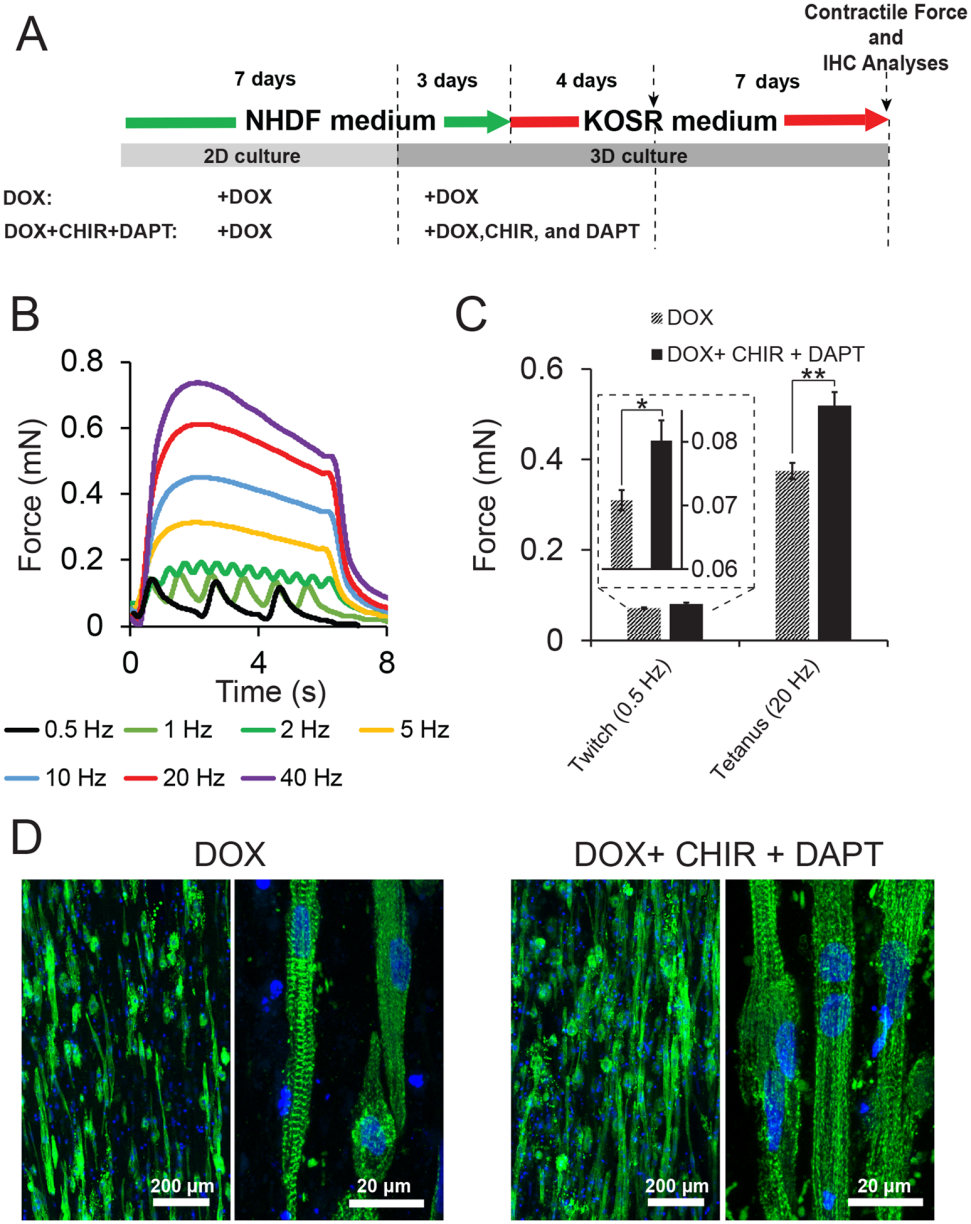
Effects of CHIR99021 and DAPT on 3D tissue constructs engineered with iMyoD-hTERT-NHDFs. (**A**) Illustration of cell culture regimens. (**B**) Representative contractile force patterns generated by the constructs cultured in the DOX + CHIR + DAPT condition illustrated in (**A**). (**C**) Treatment with CHIR99021 and DAPT enhanced functional myogenic differentiation as revealed from the comparison of the contractile forces. The experiments were conducted in triplicate with 3 independent samples. Data are presented as mean ± SEM. Statistical analysis was performed by t-test. *p < 0.05, **p < 0.01. (**D**) Longitudinal cryosections of 3D constructs stained with an anti-sarcomeric α-actinin antibody (green) and counterstained with the Hoechst 33342 nuclear marker (blue), revealing myotube alignment (from low-magnification images) and the striated structure (from high-magnification images) for both conditions.

We further reasoned that treatment with CHIR and DAPT would enhance the contractile function of the muscle constructs derived from transdifferentiated myoblast-like cells. To test this hypothesis, the culture regimen was modified: 3D culture was conducted in media supplemented with DOX, CHIR, and DAPT for 1 week (3 days in NHDF medium and 4 days in KOSR medium), followed by culture in the KOSR medium with no supplements for an additional week (the DOX + CHIR + DAPT condition in Fig. 5A). Enhancement in contractile force generation was observed: the twitch force (0.5 Hz) and the tetanic force (20 Hz) increased from 0.071 ± 0.002 mN to 0.080 ± 0.003 mN and from 0.374 ± 0.012 mN to 0.519 ± 0.034 mN, respectively, as compared with the control in which CHIR and DAPT were not added (the DOX condition in Fig. 5A) (Fig. 5B,C). Our study not only demonstrates that myogenic cells transdifferentiated from human fibroblasts via forced MyoD expression can develop into tissue constructs generating contractile forces in response to external electrical stimulation, but it also shows that contractile force generation of these constructs is enhanced by small-molecule regulators for Wnt and Notch pathways.

We further conducted immunohistochemical (IHC) analysis for the 3D constructs cultured in the two conditions illustrated in Fig. 5A. Longitudinally sectioned tissue slices stained for sarcomeric α-actinin revealed aligned myotubes in both conditions (Fig. 5D). High-magnification images showed that striated myotubes developed in both conditions, suggesting that the fibroblasts transdifferentiated into muscle cells (Fig. 5D). However, the occurrence of striated cells in these samples is not as high as that in 3D constructs engineered with primary human myoblasts^41^; and some striated patterns appear underdeveloped.

In our previous study on tissue constructs engineered with hESC-derived myogenic progenitors using the same 3D culture devices (cell seeding density at 7.5 million cells/ml), we observed twitch and tetanic forces of 0.051 ± 0.016 mN (0.5 Hz) and 0.205 ± 0.093 mN (20 Hz), respectively, at 14 days of differentiation in the KOSR medium^40^. Considering the higher cell seeding density in the current study (10 million cells/ml), the contractile forces generated by the transdifferentiated cells are similar to those generated by hESC-derived cells in the KOSR medium. However, we were able to enhance the forces generated by hESC-derived tissue constructs to 0.374 ± 0.089 mN (twitch at 0.5 Hz) and 0.748 ± 0.277 mN (tetanic at 20 Hz) at 14 days of differentiation by optimizing the differentiation medium, and these values are higher than what we observed for the transdifferentiated cells. Furthermore, the forces generated by tissue constructs engineered with primary human skeletal myoblasts (Gibco HSkM) using the same 3D culture devices (cell seeding density at 5 million cells/ml) are approximately 0.74 mN (twitch at 0.5 Hz) and 1.43 mN (tetanic at 20 Hz) (Figure S6) at 5 days of differentiation in the KOSR medium, suggesting that HSkM-derived tissue constructs can generate much higher forces than those derived from the transdifferentiated cells. These results suggest that the transdifferentiated cells differ from true muscle cells, and the method presented here has limitations and needs further improvement. Since transdifferentiated myogenic cells have several advantages over primary myoblasts or hPSC-derived myogenic cells as described in the Introduction, future studies are needed to characterize and understand the molecular, structural, and functional differences between the tissue constructs engineered with these transdifferentiated cells and those with primary myoblasts. Such studies are important because they could provide insights to improve the properties of the transdifferentiated cells and render them valuable for fundamental studies and therapy development for muscular diseases.

## Conclusion

We established the iMyoD-hTERT-NHDF cell line that could proliferate extensively and transdifferentiate into m cells upon DOX-induced MyoD expression. During myogenic differentiation of these transdifferentiated myoblast-like cells, CHIR99021, a Wnt signaling activator, promoted the commitment for terminal myogenic differentiation and DAPT, a Notch signaling inhibitor, enhanced terminal differentiation and maturation of the committed cells. Co-treatment with CHIR99021 and DAPT substantially enhanced the overall efficiency of myogenic differentiation of iMyoD-converted fibroblasts. The 3D tissue constructs engineered with iMyoD-hTERT-NHDFs generated detectable contractile forces in response to electrical stimuli after 2 weeks of 3D culture with DOX, and withdrawal of DOX in the second week of 3D culture resulted in significant increases in contractile forces, suggesting that temporally-controlled rather than prolonged MyoD expression benefits functional myogenic differentiation of these cells. These 3D constructs exhibited further enhanced contractile forces when treated with CHIR99021 and DAPT. The ability of these abundant and potentially patient-specific and disease-specific cells to develop into functional skeletal muscle constructs makes them highly valuable for many applications, such as disease modeling.

## Materials and methods

### Cell culture

Primary normal human dermal fibroblasts (NHDFs) (Gibco) were cultured in an NHDF medium composed of high glucose DMEM (Life Technologies), 20% FBS (Atlantic bioscience), 2 mM glutamax (Life Technologies), and 1% penicillin/streptomycin (Life Technologies). To confirm the robustness of the transdifferentiation method, NHDFs from two donors were used (lot numbers: 1998537 and 1165554). All NHDFs were subcultured upon 100% confluency with a 1:5 splitting ratio. For primary NHDFs, P3 to P9 were used. The Lenti-X HEK293T cell line (Takara Bio USA) was maintained in a medium composed of high glucose DMEM, 10% FBS, 2 mM glutamax, and 1% penicillin/streptomycin.

### Virus production

Lenti-X HEK293T cells were used to produce both retroviral and lentiviral vectors. Cells were seeded at 70**–**80% confluency in a T25 flask and allowed to grow overnight, and the medium was replaced with fresh medium 4 h before transfection. The cells were transfected with plasmids encoding the vector components by using the Xfect transfection reagent (Takara Bio USA) according to the manufacturer’s instruction. To produce the retroviral vector encoding hTERT, cells were co-transfected with 5 µg of the retroviral transfer plasmid pBABE-neo-hTERT (Addgene plasmid #1774)^42^, 4 µg of the packing plasmid pUMVC (Addgene plasmid #8449)^43^, and 0.5 µg of the envelop plasmid pCMV-VSV-G (Addgene plasmid #8454)^43^. To produce the lentiviral vector encoding iMyoD, cells were co-transfected with 5.6 µg of the lentiviral transfer plasmid LV-TRE-VP64 human MyoD-T2A-dsRedExpress2 (Addgene plasmid #60629)^17^, 2.7 µg of the packaging plasmid psPAX2 (Addgene plasmid #12260, a gift from Didier Trono), and 1.7 µg of the envelope plasmid pCMV-VSV-G. The medium was replaced with 4 ml of fresh medium every day, and the virus-containing media collected between 48 and 96 h after transfection were pooled and allowed to pass through a 0.45 µm sterile filter (Fisher).

### Generation of hTERT-iMyoD-NHDFs and primary iMyoD-NHDFs by viral transduction

To generate hTERT-iMyoD-NHDFs, primary NHDFs were transduced with the retroviral vector encoding hTERT and the lentiviral vector encoding iMyoD sequentially. In brief, primary NHDFs (passage 6) were seeded in a 6-well plate at 20** ~ **30% confluency and allowed to attach for 12 h, followed by replacing the medium with 1.5 ml of the medium containing hTERT retroviral vector particles and supplemented with 4 μg/ml Polybrene (Sigma-Aldrich). The medium was replaced every day with 1.5 ml of the same virus-containing medium for two additional days. The cells were then re-plated at 20** ~ **30% confluency and treated with 500 µg/ml G418 (Teknova) for 7 days to select transduced cells. The resulting hTERT-NHDFs were further transduced with the lentiviral vector encoding iMyoD in the same manner and treated with 500 ng/ml puromycin (Santa Cruz) for 5 days to select hTERT-iMyoD-NHDFs. To generate primary iMyoD-NHDFs, primary NHDFs were transduced with the lentiviral vector encoding iMyoD in the same manner and treated with 500 ng/ml puromycin for 5 days to select iMyoD-NHDFs.

The hTERT-iMyoD-NHDFs were cultured in NHDF medium containing 100 µg/ml G418 and 100 ng/ml puromycin unless stated otherwise. The iMyoD-NHDF were cultured in NHDF medium containing 100 ng/ml puromycin unless stated otherwise. Both cell types were subcultured upon 100% confluency with a 1:5 splitting ratio.

### Measurement of population doublings

The expansion capacity of hTERT-iMyoD-NHDFs and primary iMyoD-NHDFs was examined by measuring the population doubling as a function of time^29^. The starting cells were at passage 9 for both cell types. The cells were expanded in the NHDF medium (without puromycin or G418) and passaged successively for at least 20 rounds. In each round, cells were seeded in 48-well plates (2 × 10^4^ cells in each well), cultured for 3** ~ **6 days, and trypsinized for cell counting and re-plating. The experiments were conducted in triplicate. Population doublings were calculated as follows:

$$ {\text{PDL}}_{n} = \mathop \sum \limits_{i = 1}^{n} \log_{2} \frac{{N_{i} }}{{2 \times 10^{4} }} $$

where PDL_n_ is the population doubling at the *n*th round, and N_i_ is the cell number in one well at the end of the *i*th round (average of triplicates).

### Myogenic differentiation in 2D cultures

The culture regimens to drive myogenic differentiation of hTERT-iMyoD-NHDFs or iMyoD-NHDFs in 2D cultures are illustrated in Fig. 2A. Doxycycline (Sigma-Aldrich) was added in culture media at 3 µg/ml throughout the whole culture period in each regimen. This concentration was chosen because the dose–response relationship between the Dox concentration and myogenic differentiation was examined in a previous study, and 3 µg/ml was determined to be the optimal concentration^17^. Cells were seeded at 20% confluency and cultured in the NHDF medium for 7 days, trypsinized, re-plated at a density of 6 × 10^4^ cells/cm^2^, cultured in the NHDF medium for 3 days, and then cultured in the KOSR medium (Knockout DMEM (Invitrogen) supplemented with 20% knockout serum replacement (Invitrogen), 1% MEM non-essential amino acids solution (Invitrogen), 1% glutamax (Invitrogen), and 50 U/ml penicillin/streptomycin (Invitrogen))^40^ for 4 days. The differences between the culture regimens are the supplements (5 µM CHIR99021 (Sigma-Aldrich) and/or 10 μM DAPT (Tocris)) in the NHDF and KOSR media after cell re-plating: no supplement for the DOX condition; CHIR99021 for the DOX + CHIR condition; DAPT for the DOX + DAPT condition; both CHIR99021 and DAPT for the DOX + CHIR + DAPT condition. All the media contained 100 µg/ml G418 and 100 ng/ml puromycin.

### Myogenic differentiation in 3D cultures

The culture regimens to drive myogenic differentiation of hTERT-iMyoD-NHDFs in 3D tissue constructs are illustrated in Figs. 4A and 5A. Cells were seeded at 20% confluency and expanded in DOX-containing NHDF medium for 7 days in 2D culture, trypsinized, encapsulated in a hydrogel, and cast in a homemade culture device to generate 3D tissue constructs as we previously reported^40^. Each construct was generated from 195 µl of a hydrogel precursor containing the cells at a density of 10 million/ml, 6 mg/ml fibrinogen (Sigma-Aldrich), 10% (v/v) growth factor reduced Matrigel (R&D), and 1-unit bovine thrombin (Sigma-Aldrich). The hydrogel precursor was allowed to gel at 37 °C for 1 h, and the resulting 3D construct was cultured for 2 weeks. During the first week, the construct was cultured in the NHDF medium for 3 days and then in the KOSR medium for 4 days, with both media supplemented with 3 µg/ml DOX. During the second week, the KOSR medium with or without DOX was used for the two culture regimens illustrated in Fig. 4A, and the KOSR medium without DOX was used for both culture regimens illustrated in Fig. 5A. To examine the effects of CHIR and DAPT, 5 µM CHIR and 10 µM DAPT were added in the NHDF and the KOSR media during the first week of 3D culture for the DOX + CHIR + DAPT condition illustrated in Fig. 5A. All the 3D culture media contained 100 µg/ml G418 and 100 ng/ml puromycin and were supplemented with 2 mg/ml ε-aminocaproic acid (Sigma-Aldrich) to minimize scaffold degradation. Medium change was conducted every 4 days by replacing 25% of the old medium with the fresh medium.

### PCR analysis to verify viral transduction

To verify the genes encoding hTERT or MyoD were integrated into the genome of the transduced cells, their genomic DNA was extracted using the following procedures. A total of 0.1 million virally transduced NHDFs selected through antibiotic treatment were suspended in 200 µl of PBS containing 200 µg/ml RNase A (Emdmillipore) and 16 units of proteinase K (NEB) and lysed by adding 10 µl of 10% SDS. The cell lysate was mixed with 200 µl of binding buffer (4 M Guanidine HCl, 0.5 M potassium acetate, pH 4.2) and incubated at 70 °C for 10 min, followed by addition of 200 µl of ethanol. The mixture was loaded on a miniprep spin column (epoch bioscience), which was then washed with 500 µl of wash buffer I (5 M guanidine HCl, 20 mM Tris–HCl in 38% ethanol, pH 6.6) once and with 500 µl of wash buffer II (20 mM NaCl, 2 mM Tris–HCl in 80% ethanol, pH 7.5) twice. The genomic DNA was eluted with 30 µl of elution buffer (10 mM Tris–HCl, pH 8.5), and the DNA concentration and purity were determined on a spectrophotometer (BioTek Cytation 3).

Real-time PCR followed by melting curve analysis of the PCR product was conducted on a LightCycler 96 System (Roche) as previously reported^40^ to verify the presence of the genes of interest in the extracted genomic DNA. The forward and reverse PCR primers for detection of the truncated GAG domain in the pBABE-neo-hTERT plasmid, the RRE domain in the LV-TRE-VP64 human MyoD-T2A-dsRedExpress2 plasmid, and human albumin (ALB, as a reference) are shown in Table S1. For each PCR reaction, a 10 µl reaction mixture containing 1.25 ng of the extracted genomic DNA, 200 nM forward primer, 200 nM reserved primer, and 1 × LightCycler 480 SYBR Green I Master (Roche) was prepared. Each sample was pre-denatured at 95 °C for 10 min followed by 45 rounds of a 3-step temperature cycle (denaturation at 95 °C for 10 s, annealing at 54 °C for 15 s, and extension at 75 °C for 15 s). After all the 45 rounds were completed, melting curve analysis was conducted, and the presence of a gene of interest was suggested by a single sharp peak in the derivative melting curve plot indicative of specific PCR amplification. The melting temperature revealed from this plot was compared with the predicted melting temperature^44^ to further confirm the presence of the gene.

### Quantitative RT-PCR

qRT-PCR was performed to quantify the expression levels of muscle-specific genes as previously reported^40^. Briefly, the standard TRIzol-chloroform method was used to extract total RNA, and the RNA concentration and purity were determined on a BioTek Cytation 3 spectrophotometer. The RNA was treated with RQ1 DNase (Promega) and reversely transcribed into cDNA by using the Maxima First Strand cDNA Synthesis Kit (Thermofisher). RT-PCR was performed in a LightCycler 96 System (Roche) with 10 μl of reaction mixture containing 5 ng of cDNA, 200 nM forward primer, 200 nM reserved primer, and 1 × LightCycler 480 SYBR Green I Master (Roche). The thermal cycling conditions were the same as those previously mentioned (for PCR analysis to verify viral transduction). The fluorescent signal of SYBR green I was recorded after each extension step. After all the 45 thermocycles were completed, a melting curve of the PCR product was recorded to confirm the specificity of each PCR reaction. Relative gene expression normalized to the housekeeping gene β-actin (ACTB) was calculated using a standard ΔCT method. The primers are listed in Table S2.

### Immunocytochemistry and immunohistochemistry

Samples cultured in 2D were fixed with 4% paraformaldehyde (Sigma-Aldrich) for 15 min, permeabilized with 0.5% Triton X-100 for 10 min, blocked with 5% bovine serum albumin for 30 min, stained with an anti-total MHC antibody (MF20, Developmental Studies Hybridoma Bank (DSHB), 1:300 dilution) at 4 °C overnight, and then co-stained with goat anti-mouse AlexaFluor488 (Invitrogen 1:400 dilution) and the Hoechst 33,342 nuclear marker (Invitrogen 10 µg/ml) for 1 h. Each sample was washed with PBS three times (30-min incubation each time). All the procedures were conducted at room temperature unless stated otherwise. The samples were imaged on a Zeiss Axio Observer inverted microscope with a 5 × objective. The myogenic index was quantified by calculating the ratio of the number of nuclei in MF20^+^ myotubes containing at least three nuclei to the total number of nuclei in an image^19,27^. To measure the length of a myotube, a line between the two endpoints of the myotube was manually drawn along the myotube in ImageJ, and the length of the line segment was measured by ImageJ. To measure the width of a myotube, a straight line was drawn in the direction perpendicular to the local cell alignment direction, and this line crossed with the myotube surface at two points; the distance between these two crossing points was measured by ImageJ as the myotube width. For myotubes having non-uniform widths, the widths at multiple locations of each myotube representing the non-uniformity were measured. All the experiments were conducted in triplicate with 3 independent samples. For each sample, 3 non-overlapping images were acquired; for each image, at least 6 representative myotubes were analyzed.

Immunohistochemical analysis of 3D tissue constructs was conducted using our previously published method^40^. The samples were fixed with 4% paraformaldehyde. The fixed samples were treated in infiltration solution I (30% w/v sucrose and 5% w/v dimethyl sulfoxide) at 4 °C overnight, followed by in infiltration solution II (50% infiltration solution I and 50% TissueTek OCT embedding medium) at 4 °C overnight. Each sample was then embedded in OCT compound and frozen in liquid nitrogen, followed by cryosectioning longitudinally into 40 µm slices. The sections were washed with PBS for 10 min to remove excess OCT, permeabilized with 0.5% Triton X-100 for 40 min, blocked with 5% bovine serum albumin for 1 h, stained with an anti-sarcomeric α-actinin antibody (Sigma-Aldrich, 1:300 dilution) at 4 °C overnight, and then co-stained with goat anti-mouse AlexaFluor488 and the Hoechst 33342 nuclear marker for 2 h. Stained sections were mounted using Fluoromount-G (Southern Biotech) and imaged using a Zeiss LSM 700 confocal laser scanning microscope with a 10× or 63× objective. With the 63× objective, a Z-stack of 20 images and 0.5 µm Z-spacing was acquired, and the maximum intensity projection of the Z-stack is presented for each sample.

### Western blot

Cells were lysed in cold RIPA buffer (Thermoscientific) containing a protease inhibitor cocktail (Takara Bio)^45^. The total protein concentration in each lysate was determined using the BCA protein assay (Thermoscientific) according to the manufacture’s instruction. Each cell lysate (containing 40 µg of total protein) was subjected to SDS-PAGE in a 4–15% Mini-PROTEAN TGX gel (Bio-Rad) and the separated proteins were transferred to a PVDF membrane (MilliporeSigma). The membrane was blocked with the Odyssey blocking buffer (LI-COR) for 1 h, and then incubated with a primary antibody overnight at 4 °C^46^. The primary antibodies were anti-total MHC (MF20, 1:200 dilution), anti-neonatal MHC (N3.36, 1:200 dilution), and anti-actin (JLA20, 1:200 dilution), all obtained from DSHB. The membrane was washed with Tris Buffered Saline (TBS, Bio-Rad) for 30 min, and then incubated with a secondary antibody IRDye anti-mouse 680RD (LI-COR, 1:10,000 dilution) for 40 min. After washing with TBS for 30 min, blots on the membrane were visualized in a ChemiDoc Imaging System (Bio-Rad). All the antibodies were diluted in a mixture of Odyssey blocking buffer and TBS (1:1 volume ratio). All TBS contained 0.05% Tween 20 (Fisher). All the procedures were performed at room temperature unless stated otherwise.

### Measurements of contractile forces generated by 3D tissue constructs

Contractile forces generated by 3D tissue constructs in response to electrical stimuli were measured using a custom-built apparatus as previously reported^40,47,48^. Briefly, a construct was mounted on two pins in a chamber, with one pin fixed and the other adjustable and coupled to a force transducer (Harvard Apparatus, 60-2994 model). The construct was stretched by 20% of its initial length before each set of measurements. Samples were stimulated at 0.5, 1, 2, 5, 10, 20, and 40 Hz for 6 s (10 ms pulse width; 24 V) with a cardiac stimulator (Astro-Med Inc., S88 × Model), and the forces were recorded in LabView. The data were analyzed with a custom code in MATLAB. The maximum values in each twitch peak or tetanic plateau were used for quantitative analysis. Measurements of twitch forces (0.5 Hz) and tetanic forces (20 Hz) were repeated three times for each construct, and the experiments were performed in triplicate with 3 independent samples. Therefore, 27 twitch peaks and 9 tetanic force plateaus were used for quantitative analysis for each condition.

### Statistics

Data are presented as mean ± standard error of the mean. Statistical analyses were conducted by two-tailed Student’s t-tests or one-way ANOVA with Tukey’s test in GraphPad Prism 7.

## Supplementary Information

Supplementary Information

## Data Availability

All data needed to evaluate the conclusions in the paper are present in the paper and/or the Supplementary Materials. Additional data related to this paper may be requested from the authors.

## References

[1] Mercuri E, Muntoni F (2013). Muscular dystrophies. Lancet.

[2] Vandenburgh H, Shansky J, Benesch-Lee F, Skelly K, Spinazzola JM, Saponjian Y, Tseng BS (2009). Automated drug screening with contractile muscle tissue engineered from dystrophic myoblasts. Faseb J..

[3] Sharples AP, Player DJ, Martin NR, Mudera V, Stewart CE, Lewis MP (2012). Modelling in vivo skeletal muscle ageing in vitro using three-dimensional bioengineered constructs. Aging Cell.

[4] Burattini S, Ferri P, Battistelli M, Curci R, Luchetti F, Falcieri E (2004). C2C12 murine myoblasts as a model of skeletal muscle development: morpho-functional characterization. Eur. J. Histochem..

[5] Chen YW, Zhao P, Borup R, Hoffman EP (2000). Expression profiling in the muscular dystrophies: identification of novel aspects of molecular pathophysiology. J. Cell Biol..

[6] Zhu CH, Mouly V, Cooper RN, Mamchaoui K, Bigot A, Shay JW, Di Santo JP, Butler-Browne GS, Wright WE (2007). Cellular senescence in human myoblasts is overcome by human telomerase reverse transcriptase and cyclin-dependent kinase 4: consequences in aging muscle and therapeutic strategies for muscular dystrophies. Aging Cell.

[7] Mamchaoui K, Trollet C, Bigot A, Negroni E, Chaouch S, Wolff A, Kandalla PK, Marie S, Di Santo J, St Guily JL (2011). Immortalized pathological human myoblasts: towards a universal tool for the study of neuromuscular disorders. Skelet. Musc..

[8] Chua MWJ, Yildirim ED, Tan JHE, Chua YJB, Low SMC, Ding SLS, Li CW, Jiang Z, Teh BT, Yu K (2019). Assessment of different strategies for scalable production and proliferation of human myoblasts. Cell Prolif..

[9] Xu B, Magli A, Anugrah Y, Koester SJ, Perlingeiro RCR, Shen W (2018). Nanotopography-responsive myotube alignment and orientation as a sensitive phenotypic biomarker for Duchenne Muscular Dystrophy. Biomaterials.

[10] Maffioletti SM, Sarcar S, Henderson ABH, Mannhardt I, Pinton L, Moyle LA, Steele-Stallard H, Cappellari O, Wells KE, Ferrari G, Mitchell JS, Tyzack GE, Kotiadis VN, Khedr M, Ragazzi M, Wang W, Duchen MR, Patani R, Zammit PS, Wells DJ, Eschenhagen T, Tedesco FS (2018). Three-dimensional human iPSC-derived artificial skeletal muscles model muscular dystrophies and enable multilineage tissue engineering. Cell Rep.

[11] Uchimura T, Otomo J, Sato M, Sakurai H (2017). A human iPS cell myogenic differentiation system permitting high-throughput drug screening. Stem Cell Res..

[12] Darabi R, Arpke RW, Irion S, Dimos JT, Grskovic M, Kyba M, Perlingeiro RCR (2012). Human ES- and iPS-derived myogenic progenitors restore DYSTROPHIN and improve contractility upon transplantation in dystrophic mice. Cell Stem Cell.

[13] Grath A, Dai G (2019). Direct cell reprogramming for tissue engineering and regenerative medicine. J. Biol. Eng..

[14] Tapscott SJ, Davis RL, Thayer MJ, Cheng PF, Weintraub H, Lassar AB (1988). MyoD1: a nuclear phosphoprotein requiring a Myc homology region to convert fibroblasts to myoblasts. Science.

[15] Choi J, Costa ML, Mermelstein CS, Chagas C, Holtzer S, Holtzer H (1990). Myod converts primary dermal fibroblasts, chondroblasts, smooth-muscle, and retinal pigmented epithelial-cells into striated mononucleated myoblasts and multinucleated myotubes. Proc. Natl. Acad. Sci. U.S.A..

[16] Volarevic V, Markovic BS, Gazdic M, Volarevic A, Jovicic N, Arsenijevic N, Armstrong L, Djonov V, Lako M, Stojkovic M (2018). Ethical and safety issues of stem cell-based therapy. Int. J. Med. Sci..

[17] Kabadi AM, Thakore PI, Vockley CM, Ousterout DG, Gibson TM, Guilak F, Reddy TE, Gersbach CA (2015). Enhanced MyoD-induced transdifferentiation to a myogenic lineage by fusion to a potent transactivation domain. ACS Synth. Biol..

[18] Shay JW, Wright WE (2000). Hayflick, his limit, and cellular ageing. Nat. Rev. Mol. Cell Biol..

[19] Sorci G, Riuzzi F, Agneletti AL, Marchetti C, Donato R (2003). S100B inhibits myogenic differentiation and myotube formation in a RAGE-independent manner. Mol. Cell Biol..

[20] Bentzinger CF, Wang YX, Rudnicki MA (2012). Building muscle: molecular regulation of myogenesis. Cold Spring Harbor Perspect. Biol..

[21] Pavlidou T, Rosina M, Fuoco C, Gerini G, Gargioli C, Castagnoli L, Cesareni G (2017). Regulation of myoblast differentiation by metabolic perturbations induced by metformin. PLoS ONE.

[22] Tran FH, Zheng JJ (2017). Modulating the wnt signaling pathway with small molecules. Protein Sci. Publ. Protein Soc..

[23] Boularaoui SM, Abdel-Raouf KMA, Alwahab NSA, Kondash ME, Truskey GA, Teo JCM, Christoforou N (2018). Efficient transdifferentiation of human dermal fibroblasts into skeletal muscle. J. Tissue Eng. Regen. Med..

[24] Kitzmann M, Bonnielu A, Duret C, Vernus B, Barro M, Laoudj-Chenivesse D, Verdi JM, Carnac G (2006). Inhibition of notch signaling induces myotube hypertrophy by recruiting a subpopulation of reserve cells. J. Cell. Physiol..

[25] Mu X, Tang Y, Lu A, Takayama K, Usas A, Wang B, Weiss K, Huard J (2015). The role of Notch signaling in muscle progenitor cell depletion and the rapid onset of histopathology in muscular dystrophy. Hum. Mol. Genet..

[26] Choi IY, Lim H, Estrellas K, Mula J, Cohen TV, Zhang YF, Donnelly CJ, Richard JP, Kim YJ, Kim H, Kazuki Y, Oshimura M, Li HL, Hotta A, Rothstein J, Maragakis N, Wagner KR, Lee G (2016). Concordant but varied phenotypes among duchenne muscular dystrophy patient-specific myoblasts derived using a human iPSC-based model. Cell Rep..

[27] Ono Y, Sakamoto K (2017). Lipopolysaccharide inhibits myogenic differentiation of C2C12 myoblasts through the Toll-like receptor 4-nuclear factor-kappaB signaling pathway and myoblast-derived tumor necrosis factor-alpha. PLoS ONE.

[28] Attia M, Maurer M, Robinet M, Le Grand F, Fadel E, Le Panse R, Butler-Browne G, Berrih-Aknin S (2017). Muscle satellite cells are functionally impaired in myasthenia gravis: consequences on muscle regeneration. Acta Neuropathol..

[29] Chaouch S, Mouly V, Goyenvalle A, Vulin A, Mamchaoui K, Negroni E, Di Santo J, Butler-Browne G, Torrente Y, Garcia L, Furling D (2009). Immortalized skin fibroblasts expressing conditional MyoD as a renewable and reliable source of converted human muscle cells to assess therapeutic strategies for muscular dystrophies: validation of an exon-skipping approach to restore dystrophin in duchenne muscular dystrophy cells. Hum. Gene Ther..

[30] Ganassi M, Badodi S, Quiroga HPO, Zammit PS, Hinits Y, Hughes SM (2018). Myogenin promotes myocyte fusion to balance fibre number and size. Nat. Commun..

[31] Cao Y, Kumar RM, Penn BH, Berkes CA, Kooperberg C, Boyer LA, Young RA, Tapscott SJ (2006). Global and gene-specific analyses show distinct roles for Myod and Myog at a common set of promoters. EMBO J..

[32] Goh, Q., Millay, D. P. Requirement of myomaker-mediated stem cell fusion for skeletal muscle hypertrophy. *Elife* **1,** 6 (2017).

[33] Singh K, Dilworth FJ (2013). Differential modulation of cell cycle progression distinguishes members of the myogenic regulatory factor family of transcription factors. FEBS J..

[34] Hernandez-Hernandez JM, Garcia-Gonzalez EG, Brun CE, Rudnicki MA (2017). The myogenic regulatory factors, determinants of muscle development, cell identity and regeneration. Semin. Cell Dev. Biol..

[35] Lefaucheur L, Ecolan P, Lossec G, Gabillard JC, Butler-Browne GS, Herpin P (2001). Influence of early postnatal cold exposure on myofiber maturation in pig skeletal muscle. J. Muscle Res. Cell Motil..

[36] Girardi F, Le Grand F (2018). Wnt signaling in skeletal muscle development and regeneration. Progr. Mol. Biol. Transl. Sci..

[37] Koch U, Lehal R, Radtke F (2013). Stem cells living with a Notch. Development.

[38] Brack AS, Conboy IM, Conboy MJ, Shen J, Rando TA (2008). A temporal switch from Notch to Wnt signaling in muscle stem cells is necessary for normal adult myogenesis. Cell Stem Cell.

[39] Buas MF, Kadesch T (2010). Regulation of skeletal myogenesis by Notch. Exp. Cell Res..

[40] Xu B, Zhang M, Perlingeiro RC, Shen W (2019). Skeletal muscle constructs engineered from human embryonic stem cell derived myogenic progenitors exhibit enhanced contractile forces when differentiated in a medium containing EGM-2 supplements. Adv. Biosyst..

[41] Madden L, Juhas M, Kraus WE, Truskey GA, Bursac N (2015). Bioengineered human myobundles mimic clinical responses of skeletal muscle to drugs. Elife.

[42] Counter CM, Hahn WC, Wei W, Caddle SD, Beijersbergen RL, Lansdorp PM, Sedivy JM, Weinberg RA (1998). Dissociation among in vitro telomerase activity, telomere maintenance, and cellular immortalization. Proc. Natl. Acad. Sci..

[43] Stewart SA, Dykxhoorn DM, Palliser D, Mizuno H, Yu EY, An DS, Sabatini DM, Chen IS, Hahn WC, Sharp PA, Weinberg RA, Novina CD (2003). Lentivirus-delivered stable gene silencing by RNAi in primary cells. RNA.

[44] Northwestern University. Oligo Calc: Oligonucleotide Properties Calculator. http://biotools.nubic.northwestern.edu/OligoCalc.html (accessed Jan 25, 2020).

[45] Afshar ME, Abraha HY, Bakooshli MA, Davoudi S, Thavandiran N, Tung K, Ahn H, Ginsberg HJ, Zandstra PW, Gilbert PM (2020). A 96-well culture platform enables longitudinal analyses of engineered human skeletal muscle microtissue strength. Sci. Rep..

[46] Pillai-Kastoori L, Heaton S, Shiflett SD, Roberts AC, Solache A, Schutz-Geschwender AR (2020). Antibody validation for Western blot: by the user, for the user. J. Biol. Chem..

[47] Black LD, Meyers JD, Weinbaum JS, Shvelidze YA, Tranquillo RT (2009). Cell-induced alignment augments twitch force in fibrin gel-based engineered myocardium via gap junction modification. Tissue Eng. Part A.

[48] Selvaraj S, Mondragon-Gonzalez R, Xu B, Magli A, Kim H, Laine J, Kiley J, McKee H, Rinaldi F, Aho J, Tabti N, Shen W, Perlingeiro RC (2019). Screening identifies small molecules that enhance the maturation of human pluripotent stem cell-derived myotubes. Elife.

