# Supplementary material for: Functional skeletal muscle constructs from transdifferentiated human fibroblasts
Source: Sci Rep. 2020 Dec 16;10:22047. doi: 10.1038/s41598-020-78987-8 (PMC7744552; doi:10.1038/s41598-020-78987-8)
Supplement: Supplementary file 1 — Supplementary Information [file 41598_2020_78987_MOESM1_ESM.docx]

Supplementary Materials

Functional Skeletal Muscle Constructs from Transdifferentiated Human Fibroblasts

Bin Xu^1^, Allison Siehr^1^, Wei Shen^1,2,3,^*

^1^ Department of Biomedical Engineering, University of Minnesota, Minneapolis, Minnesota 55455, USA.

^2^ Stem Cell Institute, University of Minnesota, Minneapolis, Minnesota 55455, USA.

^3^ Institute for Engineering in Medicine, University of Minnesota, Minneapolis, Minnesota 55455, USA.

^*^ Corresponding author. Email: shenx104@umn.edu; Tel: +1 612 624 3771; Fax: +1 612 626 6583


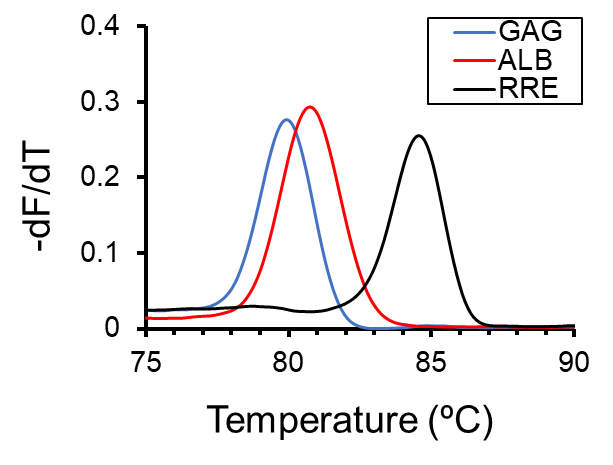


**Figure S1**. The melting curves of the PCR products of the genes encoding the truncated GAG domain, the RRE domain, and albumin (ALB, as a reference) in iMyoD-hTERT-NHDFs. The presence of GAG and RRE, which are in the pBABE-neo-hTERT and the LV-TRE-VP64 human MyoD-T2A-dsRedExpress2 plasmids, respectively, suggests successful transduction of NHDFs with hTERT and iMyoD.

**Figure S2**. CHIR99021 does not drive myogenic differentiation in the absence of MyoD expression. (A) MHC^+^ (green) myotubes were observed after iMyoD-hTERT-NHDFs were cultured in DOX-containing NHDF medium for 7 days. (B) Myotubes were absent when iMyoD-hTERT-NHDFs were cultured in DOX-free NHDF medium supplemented with CHIR99021 for 7 days. Cells were stained as shown in Figure 1C.


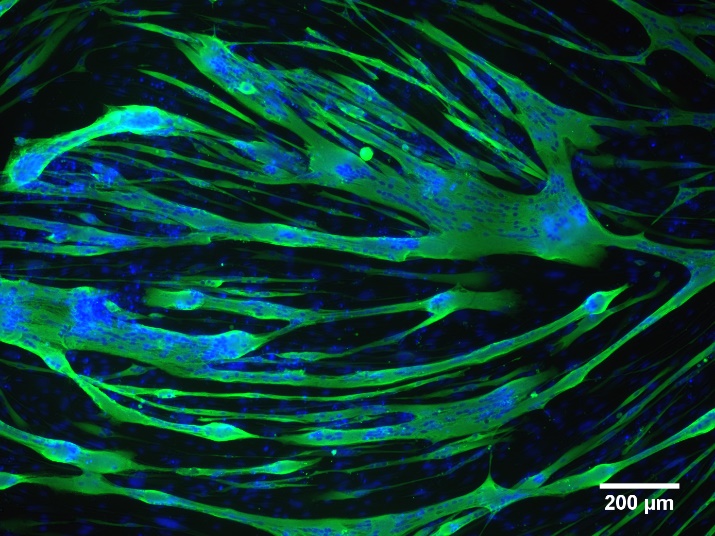


**Figure S3.** Evaluation of myogenic transdifferentiation capacity of iMyoD-hTERT-NHDFs after extensive expansion. The iMyoD-hTERT-NHDFs expanded by 15 successive passages after the cell line establishment were cultured in the DOX+CHIR+DAPT condition illustrated in Figure 2A. The cells were stained as shown in Figure 2B. The multinucleated, MHC^+^ cells possessing wide, long, and fused morphology suggest that the myogenic transdifferentiation potential was preserved.

**Figure S4.** Relative gene expression analysis for myogenic markers for primary iMyoD-NHDFs cultured using the culture regimens illustrated in Figure 2A. The expression levels were normalized to a housekeeping gene β-actin. The experiments were conducted in triplicate with 3 independent samples. Data are presented as mean ± SEM. Statistical analysis was performed by one-way ANOVA with Tukey’s test. * p<0.05, ** p<0.01.


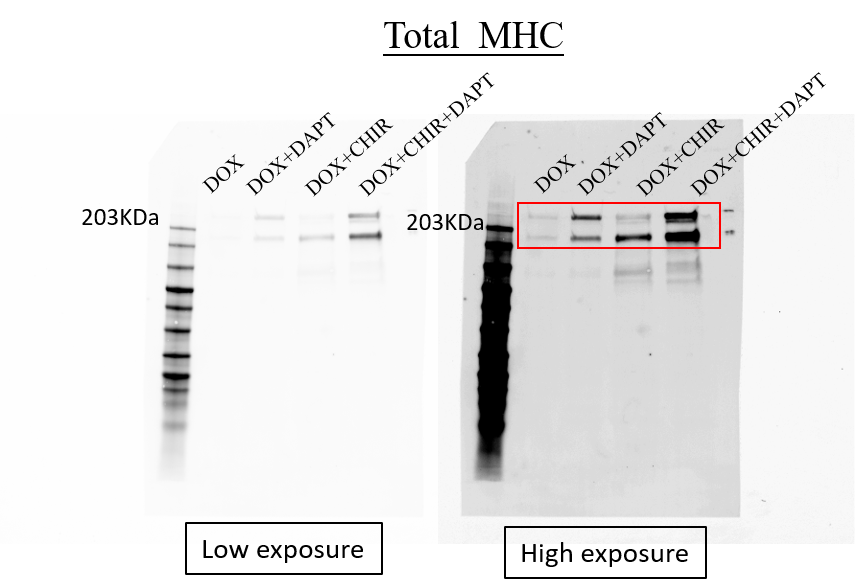


A

B


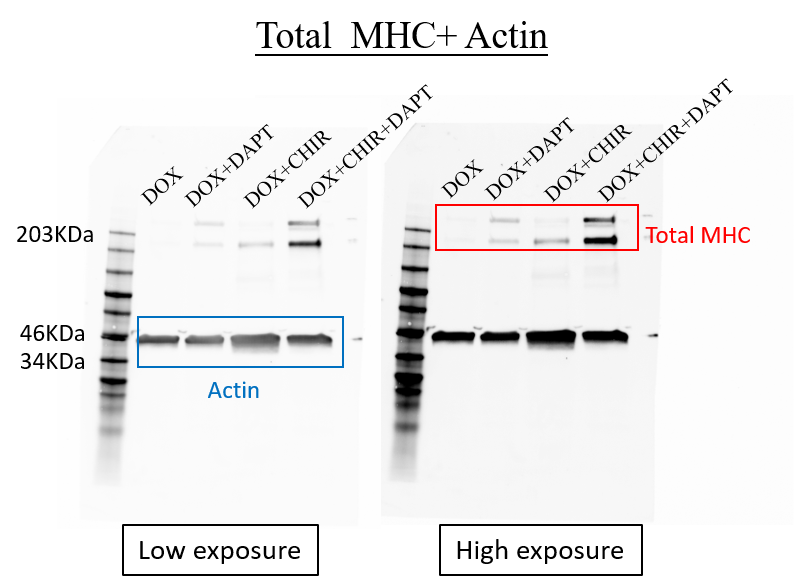


**Figure S5.** Full-length Western Blots for total MHC and neonatal MHC for cells cultured as illustrated in Figure 2A. The Western Blot data presented in Figure 3 are cropped from the full-length blots shown here. (A) Western blots for total MHC with the antibody MF20. Since MHC isoforms have molecular weights ranging from 200KDa to 250Ka (denoted in the red box), multiple bands were detected^1^. (B) The same PVDF film blotted for total MHC was further blotted for the housekeeping protein actin, which has a molecular weight of 42KDa (denoted in the blue box). (C) A second PVDF film was prepared from a gel loaded with the same amount of total proteins, and all the operational parameters were the same as those for preparing the PVDF film for blotting total MHC and actin. This PVDF film was blotted for neonatal MHC, whose molecular weight is approximately 220KDa (denoted within the red box). The bands much lower than 200KDa are non-specific bands.

C


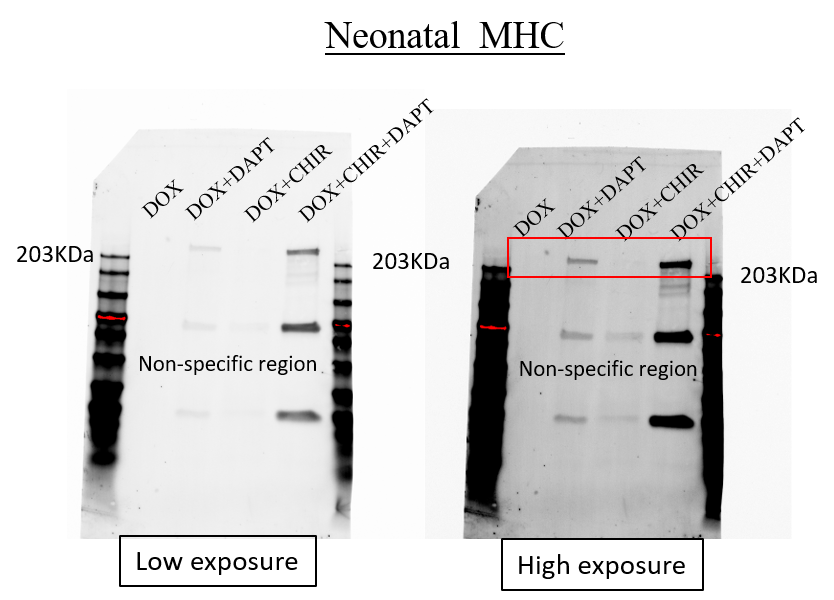


**
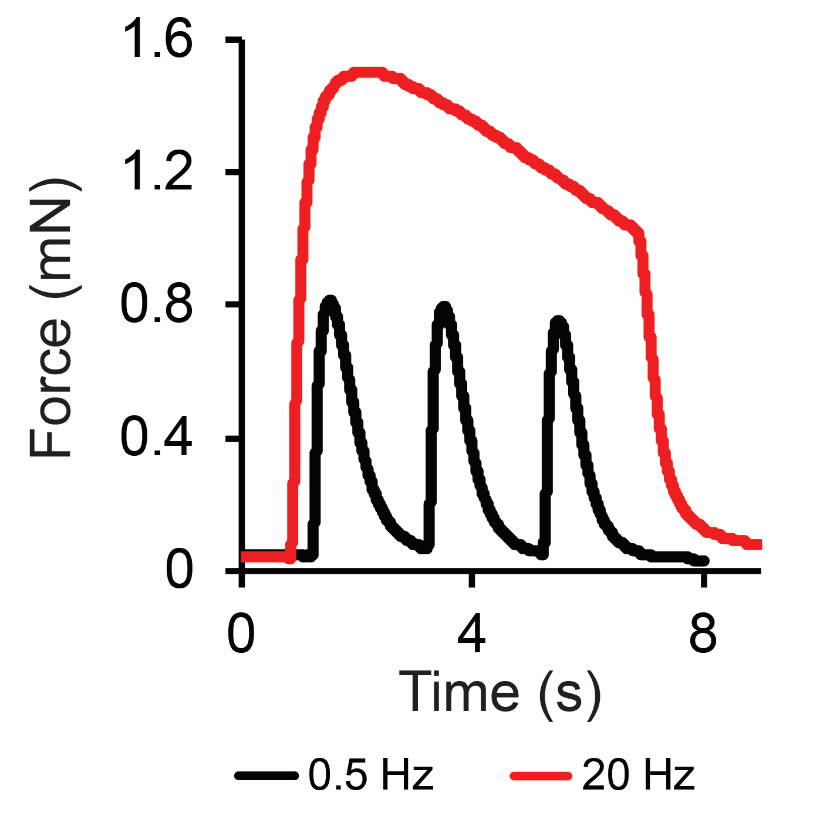
**

**Figure S6.** Representative contractile forces generated by HSkM-derived 3D constructs stimulated at 0.5 and 20 Hz. The cell seeding density was 5 million cells/ml; the constructs were cultured in the KOSR medium for 5 days.

**Table S1.** Primers to verify viral transduction

| Gene name | Primers (5’ to 3’) | PCR product length | Predicted melting temperature of the PCR product |
| --- | --- | --- | --- |
| RRE domain  (characteristic domain on VP64-MyoD vector) | Forward: GAGCTTTGTTCCTTGGGTTC  Reverse: CTCAATAGCCCTCAGCAAAT | 134 bp | 85 ºC |
| Truncated GAG domain  (characteristic domain on hTERT vector) | Forward: GGTCACTGGAAAGATGTCG  Reverse: GTAACCCAACGTCTCTTCTT | 71 bp | 80 ºC |
| ALB  (on original human genome) | Forward: GCATGCTCAAGTTGGTAGAA  Reverse: GATAGGACAGACGAAAGCAC | 94 bp | 80.4 ºC |

Predicted melting temperatures were calculated using the Oligonucleotide Properties Calculator provided by Northwestern University^2^.

**Table S2.** Primers to analyze gene expression

| Gene name | Sequence (5’- 3’) |
| --- | --- |
| ACTB | Forward: AGAAAATCTGGCACCACACC  Reversed: CCATCTCTTGCTCGAAGTC |
| ACTN2 | Forward: GAGCGCCATGAACCAGATAGA  Reversed: AGTGAAGGTCTTCCTCTGCTG^3^ |
| DAG1 | Forward: AACCCAACCAGCGCCCA  Reversed: GGGTGATATTCTGCAGGGTGATG |
| DMD | Forward: TCCTAGACCTCCTCGAAGGC  Reversed: CGCAGTGCCTTGTTGACATT^3^ |
| MYH3 | Forward: ATGGAAGTGTTCGGCATAGCT  Reversed: GGCGTACACATCCTCTGGTT |
| MYH7 | Forward: TGCCACATCTTGATCTGCTCAG  Reversed: TTGCTTTATTCTGCTTCCTCCCA |
| MYH8 | Forward: TCTTCTGGAAGAAATGAGAGATGA  Reversed: GCTTCTCTCCTTTGCAACATC |
| MYOG | Forward: CAGGGGATCATCTGCTCACG  Reversed: TGGGCATGGTTTCATCTGGG |
| MYMK | Forward: CGTCACGACATCCTGGAGTA  Reversed: TTGACCTCTTGGGTTCGTCG^3^ |
| TNNI1 | Forward: CGAGAGAGATGACCCCTTGC  Reversed: AAACGTATGCGTGTCCTGGT |

Reference:

1. Lefaucheur, L.; Ecolan, P.; Lossec, G.; Gabillard, J. C.; Butler-Browne, G. S.; Herpin, P., Influence of early postnatal cold exposure on myofiber maturation in pig skeletal muscle. Journal of Muscle Research and Cell Motility 2001, 22 (5), 439-452.
2. Northwestern University. Oligo Calc: Oligonucleotide Properties Calculator. http://biotools.nubic.northwestern.edu/OligoCalc.html (accessed Jan 25,2020).
3. Kabadi, A. M.; Thakore, P. I.; Vockley, C. M.; Ousterout, D. G.; Gibson, T. M.; Guilak, F.; Reddy, T. E.; Gersbach, C. A., Enhanced MyoD-induced transdifferentiation to a myogenic lineage by fusion to a potent transactivation domain. ACS synthetic biology 2015, 4 (6), 689-99.
